# Usefulness of the Round Endcap Expandable Cage Placed on the Vertebral Ring Apophysis in Anterior Spinal Reconstruction

**DOI:** 10.7759/cureus.23586

**Published:** 2022-03-28

**Authors:** Shun Okuwaki, Masaki Tatsumura, Fumihiko Eto, Toru Funayama, Masashi Yamazaki

**Affiliations:** 1 Department of Orthopedic Surgery, Faculty of Medicine, University of Tsukuba, Tsukuba, JPN; 2 Department of Orthopedic Surgery, Mito Kyodo General Hospital, Mito, JPN

**Keywords:** osteoporotic vertebral fracture, vertebral ring apophysis, anterior reconstruction, round endcap expandable cage, thoracolumbar vertebral fracture

## Abstract

Rectangular endcap expandable cages are common in anterior thoracolumbar spine restoration. However, the cage is often too large to place in small, elderly women. In this study, we evaluated a method to place a round endcap expandable cage on the vertebral ring apophysis in elderly women. From April 2017 to August 2020, five women (mean age 75.8 years) underwent anterior-posterior spinal fusion with a round endcap expandable cage on the vertebral ring apophysis at the thoracolumbar junction. The local kyphotic angle, coronal Cobb angle, and intervertebral height were evaluated pre-and postoperatively. Cage subsidence and bone union were evaluated. The mean local kyphotic angle, coronal Cobb angle, and intervertebral height before surgery were 35.2°, 10.0°, and 65.3 mm, respectively. Immediately postoperatively, 1 week and 3 weeks after surgery, the kyphotic angle was 13.4°, 16.6°, and 18.5°; coronal Cobb angle was 2.8°, 2.2°, and 4.3°; and intervertebral height was 76.2 mm, 71.8 mm, and 70.6 mm. Cage subsidence was not observed and the bone union was achieved in all cases. An expandable cage with a round endcap was placed in small, elderly women by inserting the cage over the strong apophysis of the vertebral body. This technique may be useful to reduce the risk of postoperative subsidence and correction loss.

## Introduction

Anterior column reconstruction with posterior fixation using an expandable cage is commonly used for patients with osteoporotic vertebral fracture (OVF) in the elderly [1,2]. A rectangular endcap with a wide footprint that contacts the vertebral ring apophysis has a larger contact area and better surgical outcomes than the round endcap [3,4].

However, there are some cases in which the rectangular endcap expandable cage is excessively large and difficult to place in small, elderly women. Furthermore, in these patients, osteoporosis is severe, and if the rectangular expandable cage is forced into place, there is a high risk of loss of correction due to intraoperative endplate damage and postoperative cage subsidence. In these cases, a smaller round endcap expandable cage often can be inserted. When using round endcap cages, the risk of postoperative subsidence is higher because of a smaller footprint [5].

In this study, we evaluated a technique to replace a round endcap expandable cage in contact with the vertebral ring apophysis on one side of the vertebral body, which is considered to have high mechanical strength, instead of the center of the vertebral body, to reduce postoperative cage sinking and endplate damage.

## Case presentation

**Figure 1:**
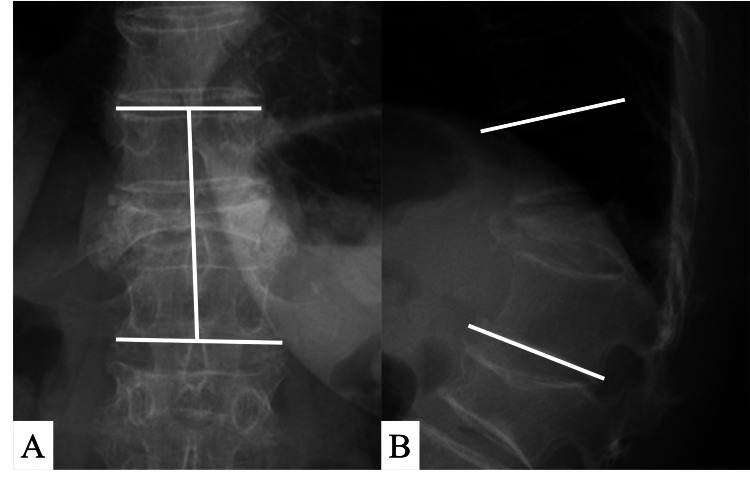
Radiologic parameters. A: The coronal Cobb angle was measured at the angle between the intact endplates cranial and caudal to the fractured vertebra on an anterior-posterior radiograph. The intervertebral height was measured between the anterior mar­gins of the intact endplates cranial and caudal to the fractured vertebra. B: The kyphotic angle on a lateral radiograph was measured by calculat­ing the angle of the intact endplates cranial and caudal to the frac­tured vertebra to evaluate the mean sagittal correction.

Between April 2017 and August 2020, five consecutive patients with OVF at the thoracolumbar junction were treated with combined anterior-posterior fusion with vertebrectomy and expandable cage placement for pseudarthrosis or delayed collapse. All patients were women over 70 years who underwent surgical intervention under circumstances that indicated mechanical instability, progressively worsening neurological status, and/or back pain unresponsive to nonsurgical treatment including parathyroid hormone and wearing a brace. For all patients, conventional radiography, computed tomography (CT), and magnetic resonance imaging (MRI) were conducted preoperatively. Patient data, including age, height, body weight, level of vertebrectomy, bone mineral density (BMD) (measured by dual-energy X-ray absorptiometry at the femoral neck), the total surgical time, and blood loss were recorded. The local kyphotic angle and coronal Cobb angle were measured radiologically by calculat­ing the angle of the intact endplates cranial and caudal to the fractured vertebra to evaluate the mean sagittal and coronal correction. Also, intervertebral height was measured between the anterior margins of the intact endplates cranial and caudal to the fractured vertebra to estimate subsidence before surgery and at the final follow-up using a plain radiograph (Figure 1) [6].

We evaluated these parameters preoperatively, postoperatively, and after 1-year and 3-year follow-ups. One patient missed measurement at the 3-year follow-up. Subsidence was measured as the difference between postoperative 1-year and 3-year follow-ups. We defined endplate injury as the cage subsiding by 5 mm or more immediately after surgery to assess instrumentation-related adverse events. Bone union was evaluated by the extent of continuous trabecular formation between the vertebral bodies or bony bridging outside the vertebral bodies on CT.

Patient demographics are shown in Table 1.

**Table 1:** Patient demographic data

Case	Age (years), Sex	Height (cm)	BMI (m/kg^2^)	BMD (g/cm^2^)	Vertebra fractured	Range of fusion	Follow-up periods (months)	Operative time (min)	Blood loss (ml)	Period for bone union (months)
1	75, F	148	17.6	0.555	T12	T10-L2	48	429	399	12
2	81, F	143	26.4	0.727	L2	T12-L4	48	416	963	18
3	75, F	148	22.1	0.699	T12	T9-L3	42	513	356	11
4	72, F	141	18.1	0.562	L1	T10-L4	36	472	612	23
5	76, F	155	13.9	0.588	L1	T11-L4	18	454	388	12

**Figure 2:**
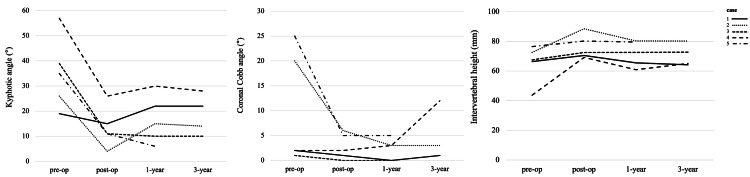
Change in the kyphotic angle (left), the coronal Cobb angle (middle), and the intervertebral height (right).

The mean age was 75.8 years (range: 72-81 years), height was 147 cm (range: 140-155 cm), and BMD was 0.63 g/cm^2^ (range: 0.56-0.73 g/cm^2^). The mean duration of follow-up was 38.4 months (range: 12-48 months), operating time was 456.8 minutes (range: 416-513 minutes), and blood loss was 543.6 ml (range: 356-963 ml). The mean local kyphotic angle was 35.2° before surgery, 13.4° immediately after surgery, 16.6° at 1-year follow-up, and 18.5° at 3-year follow-up. There was a correction loss of 5.1° from immediately after surgery to final observation. The mean coronal Cobb angle was 10° before surgery, 2.8° immediately after surgery, 2.2° at 1-year follow-up, and 4.3° at 3-year follow-up. The mean intervertebral height was 65.3 mm before surgery, 76.2 mm immediately after surgery, 71.8 mm at 1-year follow-up, and 70.6 mm at 3-year follow-up (Figure 2).

Cage subsidence of 5 mm or more was not observed in any patient. Bone union was observed in all patients in whom CT evaluation could be performed postoperatively.

Surgical procedure

Initially, posterior fixation was performed 2 or 3 levels above and 2 or 3 levels below the fracture with pedicle screws to achieve a reduction of kyphosis. A second surgery was performed one week after the first surgery. The patient was placed in the lateral position, and the expandable cage was inserted in the cavity after subtotal vertebrectomy with a sufficient amount of autologous bone transplantation. Usually, fractures were ap­proached using the retroperitoneal and transpleural routes. All patients underwent implantation of a round endcap expandable cage (SynCage-EX; Synthes, Stratec Medical, Oberdorf, Switzerland). For resection of T12 through L2, the smallest cages were 20 mm tall with -5 °endcaps, or 23 mm tall with 0° endcaps. The implant was filled with a combination of locally harvested bone before being positioned in the vertebrectomy space, and rib bone was packed into the right side of the vertebral endplate. The cage was placed in contact with the vertebral ring apophysis on the left side of the vertebral endplate, not in the center as is conventional.

The patients were allowed to leave the bed using a rigid brace two days after surgery. The rigid brace was used for six months postoperatively. All patients were treated with parathyroid hormone, which was continued until the bone union was achieved.

Representative case

**Figure 3:**
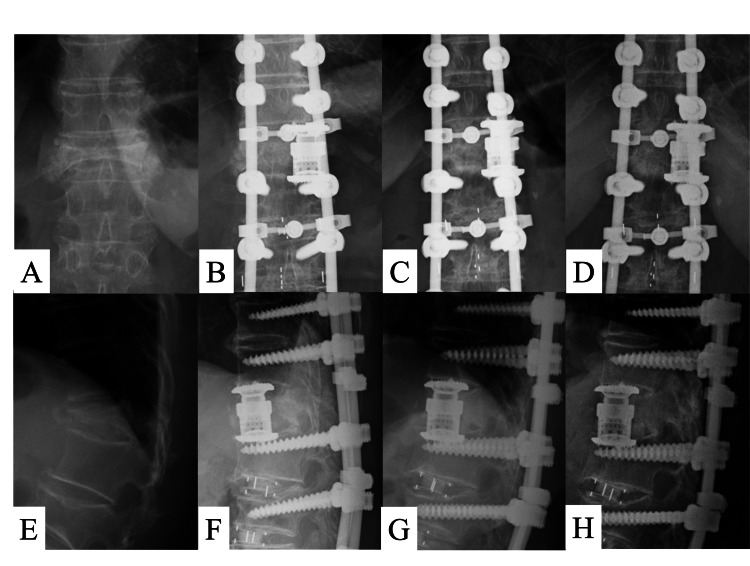
Representative case. The case of a 75-year-old woman with T12 vertebral burst fracture. A-D) Preoperative, postoperative, 1-year follow-up, and 3-year follow-up anterior-posterior radiographs. E-H) Preoperative, postoperative, 1-year follow-up, and 3-year follow-up lateral radiographs.

A 75-year-old woman underwent a combined anterior-posterior spinal fusion with a round endcap expandable cage for vertebral pseudarthrosis after OVF at T12. The operating time was 513 minutes (253 minutes during the first posterior surgery and 260 minutes during the second anterior surgery), with blood loss of 356 ml (160 ml at the first posterior surgery and 196 ml at the second anterior surgery). No blood transfusion was required, and there were no complications during the perioperative period. The patient started to mobilize one week after surgery and wore a rigid brace for six months postoperatively. Bone union was observed 11 months after surgery without any instrumentation-related events. The local kyphotic angle improved from 39° before surgery to 11° immediately after surgery, to 10° at both 1-year and 3-year follow-ups. The coronal Cobb angle changed from 1° before surgery to 0° immediately after surgery and through 1-year follow-up, to 1° at 3-year follow-up. The intervertebral height improved from 67.5 mm before surgery to 72.5 mm immediately after surgery, 72.6 mm at 1-year follow-up, and 72.7 mm at 3-year follow-up (Figure 3).

## Discussion

The present study using a round endcap expandable cage on the vertebral ring apophysis improved the local kyphotic angle and increased intervertebral height. The coronal Cobb angle was not worse after surgery. There were no cases of cage subsidence of 5 mm or more and all patients achieved bone union.

Expandable cages offer an adjustable size and tight press-fit fixation of the endplates, achieving great stability of the spine. However, there are cases where rectangular endcap expandable cages cannot be used due to the limited size of devices that are currently approved and available. In the rectangular endcap expandable cage, the minimum standard cephalocaudal length is 27 mm. A previous observational study of vertebral body height in Poland showed that in the thoracolumbar transitional region, the median vertebral body height of Th11 was 23.71±2.04 mm [7]. In that report, the mean age of the women was 67.9 years, and their height was approximately 160 cm. In the present study, the mean age was 75.8 years, and the mean height was 147 cm; it was expected that the vertebral height would be smaller in small, elderly women. A rectangular endcap expandable cage may be too large when used in elderly women. Overcorrection of anterior fixation should be avoided because it destroys the endplate cartilage and causes dislocation of the implant. When anterior fixation is used in such cases, the options are to use the smaller round endcap expandable cage placed in the cephalocaudal direction. The concern with using a round endcap expandable cage is postoperative subsidence [3]. Previous cadaver studies reported that the strength of the vertebral endplate is higher at the vertebral ring apophysis than at the center, posterior than anterior, L5 than L1, and inferior to superior endplates [8,9]. Therefore, we devised a method to apply a small round endcap expandable cage with a small contact area to the vertebral ring apophysis with high strength. There were no major complications during the postoperative observation period, suggesting that this technique may be useful.

In this report, the number of patients was small and there were no controls. In the future, it will be necessary to increase the number of cases and evaluate long-term results.

## Conclusions

An expandable cage with a round endcap was placed in small, elderly women by inserting the cage over the strong apophysis of the vertebral body. This technique may be useful to reduce the risk of postoperative subsidence and correction loss.
